# A Promise Generously Fulfilled

**Published:** 1885-06

**Authors:** 


### A Promise Generously Fulfilled.

The publication of a translation of Lumssen's great work on Special Pathology and Therapeutics involved an enormous financial responsibility, and could succeed only by the liberal support of the profession. Messrs. Wm. Wood & Co. have always acted upon the belief that the American physician wanted, and was willing to pay for, the best productions of either native or foreign talent. Determining, therefore, upon the publication of this great work, the price was fixed upon the basis of fifteen volumes of 500 to 700 pages each. It was soon found, however, that new matter and material would swell the original estimate greatly, and the American publishers found that they had but two alternatives, either to increase the number of volumes or the number of pages in each. The first would throw the additional expense upon the subscriber, the second upon themselves. With remarkable generosity, they chose the latter alternative, thereby giving their subscribers about fifty per cent. more matter than the original announcement promised. Notwithstanding this increase, the chapters on diseases of the skin were omitted from the work, for the very good reason that the articles were not written until quite recently. That no one might have any reason to complain of their failing to receive "Diseases of the Skin," Messrs. Wood & Co. announced at that time that when the articles appeared in Germany they would promptly have them translated and publish them, and present them to each subscriber to Lumssen who had completed his set. This promise they are now ready to fulfill, and we confess that we are agreeably surprised at the unprecedented liberality in its fulfillment. The new volume, in shape and general appearance, is somewhat different from ordinary medical publications, to distinguish it as a book prepared expressly for presentation. It contains 658 pages, and comprises contributions from some of the most distinguished dermatologists of the German school. The illustrations are excellent, many of them colored, and the publishers have plainly not spared expense in the issue of the volume. In a communication to this journal, they request us to notify all the subscribers to "Lumssen" to send to them their present address, and also their address at the time of their subscription.

---

Dr. Floyd S. Crego, who for several years has filled the position of Second Assistant Physician at the Buffalo State Insane Asylum, has resigned that position with a view to a permanent settlement in Buffalo for the practice of medicine. The Asylum loses, in the retirement of this accomplished and talented official, the services of an experienced medical attendant, who has combined with superior natural gifts, the advantages of broad intellectual and professional culture. Dr. Crego's success, in the public position from which he retires with the professional prestige earned through assiduous labor, foreshadows the professional position to which his superior abilities and attainments will surely gain for him at an early date.

Dr. Crego's connection with the Medical Department of Niagara University as lecturer on nervous diseases will, we hope, turn his attention especially to this important department of medicine. Here, with large experience and familiarity with a class of diseases prevalent in this community, we are confident he will find ample opportunity to employ the practical knowledge gained in the excellent institution which regretfully loses his services.

